# Effects of Parents’ Level of Education and Economic Status on the Age at Cochlear Implantation in Children

**Published:** 2012

**Authors:** Zahra Jeddi, Zahra Jafari, Masoud Motasaddi Zarandy

**Affiliations:** 1 Department of audiology, Faculty of Rehabilitation, Tehran University of Medical Sciences, Tehran, Iran; 2 Department of Basic Sciences in Rehabilitation, Rehabilitation Research Center, Faculty of Rehabilitation, Tehran University of Medical Sciences, Tehran, Iran; 3 Cochlear Implant Research Center, Amir-Alam Hospital, Faculty of Medicine, Tehran University of Medical Sciences, Tehran, Iran

**Keywords:** Age, Cochlear implantation, Hearing loss, Diagnosis, Economic status, Educational level

## Abstract

**Introduction:**

Cochlear implantation can facilitate the development of communication skills in children with profound hearing loss. The objectives of our study were to determine the average ages at suspicion and diagnosis of hearing loss, amplification, intervention, and performing the cochlear implantation and to investigate the effects of the parents’ level of education and economic circumstances on the age of the child at cochlear implantation.

**Materials and Methods:**

The parents of 96 children with profound sensorineural hearing loss who had received a cochlear implant at Amir-Alam Cochlear Implant Center between 2008 and 2010 were asked to complete a survey. The survey included demographic information, and birth, medical, and hearing loss history of their child. Study data were obtained through the patient database in the Cochlear Implant Center and interviews with the parents.

**Results:**

The mean times between the age of the children at diagnosis of hearing loss and amplification, beginning the rehabilitation program, and performing the cochlear implantation were 4.05 (±0.86), 2.59 (±0.9), and 25.43 (±1.45) months, respectively; delays that were statistically significant (P≤0.004). In 47.9 percent of cases, the parents were the first people to suspect the occurrence of hearing loss in their child. Statistical analysis indicated that the age at cochlear implantation decreases as the educational level of the parents increases (P≤0.003). There was also a significant difference between parents’ economic circumstances and the age of cochlear implantation (P<0.0001).

**Conclusion:**

There is still a remarkable delay between the diagnosis of hearing loss and aural rehabilitation in hearing-impaired children. Parents’ levels of education and economic circumstances have a noticeable effect on the age of cochlear implantation in hearing-impaired children.

## Introduction

The consequence of late identification of hearing loss manifests as poor auditory, language, cognition, and psychosocial skills, which prohibits academic achievement and ultimately results in poor vocational prospects (1). 

In addition, delay in the diagnosis of hearing loss can be a cause of anxiety and stress for the family of hearing impaired children (2). Newborn hearing screening children are an essential means of identifying hearing loss in early childhood (3). The overall success of newborn hearing screening and their long-term effects on the development of communication skills of hearing impaired children depends on the knowledge and attitude of parents toward hearing loss (4). The age of identification of hearing loss is also affected by the socioeconomic circumstances of the family (3). 

Early identification of hearing loss is the most important step for attaining successful communication outcomes in deaf children and leads, in turn, to earlier intervention and decreases in the age at which cochlear implantation is performed (5). Similarly, family characteristics and their involvement in the rehabilitation process can reduce the negative effects of late diagnosis of hearing loss (6). Nowadays, it is known that early cochlear implantation enables deaf children to achieve age-appropriate developmental skills (7).

Several studies have considered the factors that influence the age at diagnosis of hearing loss and any consequent intervention in various parts of the world. Van der Spuy and colleagues, in their study in South Africa, reported significant delays in the diagnosis of hearing loss and intervention. These delays were attributed to inadequate support services for early intervention in this country (8). 

A study by Gopal and colleagues mentioned the long time lapse between identification of hearing loss and the fitting of hearing aids (9). Watkin and colleagues indicated that identification and verification of hearing loss occurs at a lower mean age in deaf children who are identified through newborn hearing screening compared with children who do not receive newborn hearing screening. The authors also highlighted the worth of a follow-up process after newborn hearing screening have been performed (10). Similarly, a study by Danhauer reported that the age at the start of rehabilitation decreases as screening programs become more established (11). 

Ozcebe and colleagues suggested that poor socioeconomic circumstances and a low level of knowledge in a family contribute to late identification of hearing loss and intervention (3). On the other hand, Fitzpatrick and colleagues suggested that the late identification of hearing loss might have a lesser effect in families with a higher level of education (6). 

In terms of treatment outcomes, the results of several studies suggest that children who are diagnosed early and receive early amplification, intervention, and cochlear implantation, may acquire developmental skills equivalent to their hearing peers (12-14). Sevinc and colleagues concluded that the age of cochlear implantation is an important factor for speech production skills (15). Nicholas and Geers indicated that spoken language scores decrease as the age at cochlear implantation increases (16). Lester and colleagues highlighted the necessity of early intervention and referral to a cochlear implantation center in order to reduce the age at which the cochlear implantation is performed (17). 

Given the importance of early identification of hearing loss for the development of communication skills and the role of family in diagnosis and intervention in hearing loss, the purpose of this study was to: 1) determine the average ages at suspicion and diagnosis of hearing loss, amplification, intervention, and cochlear implantation; and 2) investigate the effect of parents’ level of education and economic circumstances on the age at which cochlear implantation is performed in deaf children. The results of this study can inform experts about the diagnosis and treatment of hearing loss in Iran. Moreover, a survey of the ages at which the various stages of intervention are performed and the cause of delays between them is a key to resolving the problems related to the performance of cochlear implantation in Iran.

## Materials and Methods

*Participants*

Our study sample consisted of 96 children under 6 years of age with profound sensorineural hearing loss (boys: 57.3 percent and girls: 42.7 percent; average age: 42.15 (±11.00) months) who had received cochlear implantation in Amir-Alam Cochlear Implantation Center in Tehran between 2008 and 2010 and their parents. 

*Procedure*

The parents of the deaf children who had undergone cochlear implantation all gave their informed consent to be included in the study. Data were collected from a review of the cochlear implantation center patient database and interviews with the parents. A 31 item survey designed for the interviews with the parents included: demographic information (7 items), the child’s birth history (11 items), medical history (2 items) and hearing loss history (11 items).

*Data analysis*

Significant differences between the ages of the children at the different stages of intervention studied were determined by Student’s independent t-test. The effect of the parents’ level of literacy and economic circumstances on the age of the child at cochlear implantation was examined using the Wilcoxon test. Student’s independent t-test was used to determine the effect of family history of hearing loss and the effect of the sex of the children on the ages at which they were diagnosed and received the various interventions. Significant differences between the number of family members and the studied ages were analyzed using Pearson's correlation test. Data analysis was performed using SPSS 16.0 and *P*<0.05 was considered to indicate a significant difference between the data. 

## Results

The average ages at suspicion of hearing loss, diagnosis of hearing loss, amplification, beginning the rehabilitation program, performing the cochlear implantation and beginning the use of the cochlear implant were 6.73 (±5.8), 9.35 (±5.79), 13.41 (±6.11), 16 (±6.37), 41.25 (±11.12) and 42.15 (±11.00) months, respectively. Consequently, the average delays between the ages at suspicion and diagnosis, amplification, beginning the rehabilitation program, and cochlear implantation operation were 2.62 (±0.84) (*P*=0.002), 4.05 (±0.86) (*P*<0.0001), 2.59 (±0.9) (*P*=0.004), 25.43 (±1.45) months (*P*<0.0001), respectively (see Table 1), and there were statistically significant differences between them. The mean duration of pre-implant hearing aid use was 27.86 (± 8.75) months.

**Table 1:** Statistical analysis of the delays between the studied ages

**Delay between** ** the ages **	**Suspicion and** **diagnosis of HL**	**Diagnosis and** **amplification**	**Amplification and** **rehabilitation**	**Rehabilitation and CI**
**Mean ± SD**	2.62 (±0.84)	4.05 (±0.86)	2.59 (±0.9)	25.43 (±1.45)
**P-value**	0.002	< 0.0001	0.004	< 0.0001

HL= hearing loss; CI= cochlear implantation

A total of 43.8 percent of the deaf children were identified through newborn hearing tests. In 47.9 percent of the children their parents were the first people to suspect the occurrence of hearing loss in their child. Consanguinity was observed in 68.8 percent of the families, including 48 cases of marriage between first cousins and 18 cases between second cousins. 

**Fig 1:**
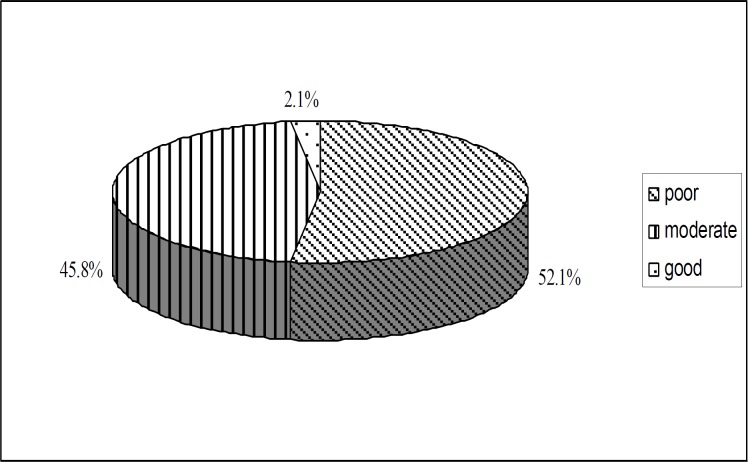
Distribution of the economic circumstances of the families

According to the parent interviews, 52.1% of the parents had poor economic circumstances, 45.8% had moderate economic circumstances and 2.1% had good economic circumstances (Fig.1). Statistical analysis indicated that the age at cochlear implantation decreased as the level of the parents’ economic circumstances increased (*P*<0.0001).

The distribution of the parents’ levels of education is shown in Table 2. Analysis indicated that there was a significant decrease in the age at cochlear implantation with an increase in the level of education of the children’s fathers (P=0.003) and mothers (P<0.0001).

**Table 2:** Distribution of the parents’ level of education

**Educational level**	**Percent **	
	**Father**	**Mother**
Under diploma	51	49
Diploma	40.6	29.9
Post diploma	2.1	9.4
B.S.	6.2	12.5

Risk factors associated with hearing loss were reported in 51% of cases. Hyperbillirubinemia (26%), family history of congenital sensorineural hearing loss (26%), and pre- and post-term births (2.1 and 3.1%, respectively) were the most frequently reported risk factors. Statistical analysis revealed that deaf children with a family history of congenital sensorineural hearing loss had lower mean ages of suspicion (*P*<0.0001) and diagnosis (*P*=0.012) compared to those without such family history.

The number of children in a family ranged 1 to 7. There were no significant differences between the number of family members and the ages at diagnosis and during the various stages of rehabilitation program (*P*≥0.319). Moreover, there were no significant differences between the sexes of the deaf children and the studied ages (*P*≥0.077).

## Discussion

A delay between the ages of suspicion and diagnosis of hearing loss was observed in this study, as has previously been reported by Lotfi (18), Jafari (19), Prendergast (20), and Ozcebe (3). According to the statements of some mothers, denial of a child's hearing loss by parents and other family members prohibits referral to a physician and thus results in a delay in diagnosis. The mean age of diagnosis in our study was 9.35 months. In several studies in the literature the age at diagnosis of hearing loss has been reported as being younger. Dalzell and colleagues reported the median age at diagnosis to be 3 months (21). Similarly, Russ and colleagues indicated that the mean age at diagnosis was 6 months (22), while Danhauer and colleagues showed that the age at diagnosis was less than 1.5 months (11). Possible reasons for a lower age at diagnosis in these studies compared with our study could be failure to implement newborn hearing screening widely and also a lack of public awareness about hearing loss symptoms and the importance of early identification of hearing loss in Iran. 

The mean age at diagnosis of hearing loss in the present study is close to the results of a study by Kennedy and colleagues where the median age at diagnosis and amplification was reported to be 10 and 15 months, respectively (14). This could be attributed to increasing levels of knowledge of parents, physicians and other people about the importance of early intervention for children with sensorineural hearing loss. However, there is still a noticeable delay between diagnosis of hearing loss and amplification, as has been pointed out by Kennedy (14), van der Spuy (8) and Gopal (9). Delays in fitting amplification devices may be due to the high cost of hearing aids and a lack of insurance. Final diagnosis of hearing loss with behavioral and electrophysiological tests in the public healthcare system is also a lengthy administrative process. Moreover, the time between the parents’ suspicion and hearing loss diagnosis by an audiologist and physician increases the time span of this process. 

On the other hand, even children with early amplification may not receive timely intervention programs. The average time interval between amplification and intervention in our study was 2.59 months, which is lower than the 6.5 months reported by Ozcebe and colleagues (3). This has been attributed to the time interval between these two studies and increased public awareness about hearing loss and the importance of early intervention in recent years. Counseling families of deaf children about the importance of early intervention for development of communication skills may lead to a smaller delay between age at diagnosis and age at intervention.

We observed noticeable differences between the age at amplification and age at referral to a cochlear implantation center, and also, the age on beginning the rehabilitation program and age at cochlear implantation. Fitzpatrick and colleagues, in their study on parents’ perspectives of the impact of the early diagnosis of hearing loss, reported that parents of children with cochlear implants commented that the child would not have received cochlear implantation until one year of age in spite of the age at identification of hearing loss (2). The possible reasons for delays in performing cochlear implantation in Iran could be due to the limited number of cochlear implantation centers and external policies related to the import of cochlear implants into the country. Another reason for the delay could also be a low level of awareness regarding cochlear implantation and the effect of age at implantation on skill development in deaf children.

**Figure 2:**
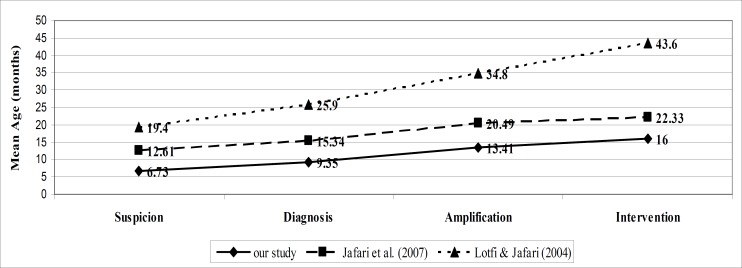
Comparison of the ages of children at suspicion, diagnosis, amplification, and intervention between this study and other studies

Our study revealed remarkable improvements in the ages at suspicion, diagnosis, amplification, and intervention compared to previous studies in Iran performed by Lotfi and Jafari (18) and Jafari and colleagues (19) in 2004 and 2007, respectively (Fig. 2). 

Reduction in the studied ages were also reported in the Durieux-Smith (23), Francois (24), and Ozcebe (3) studies. This may be due to an increase in the implementation of newborn hearing screening over time. In addition, developments in technology, pediatric diagnostic tests, and rehabilitation intervention services over time could have resulted in earlier and easier diagnosis and treatment of hearing loss and, therefore, decreases in the ages at which these occur.

Overall, 47.9 percent of our parents suspected the presence of hearing loss in their child, while 43.8 percent of our children were identified through newborn hearing screening. Watkin and colleagues reported identification of hearing loss as a result of parental suspicion and newborn hearing tests to be 54 and 46 percent, respectively. The authors suggested that the age of identification of deafness is dependent on newborn hearing screening and parental suspicion. They also suggested that approaches to improving both screening programs and parental suspicion are required (25).

A family history of congenital sensorineural hearing loss is one of the risk factors for hearing loss which may reduce the age at suspicion and identification of hearing loss. Nevertheless, the presence of hearing loss in other family members had no important effect on the ages at amplification, intervention, and cochlear implantation in deaf children in this study. In a study by Durieux-Smith and colleagues, children who had risk factors for hearing loss were diagnosed and amplified earlier than children without risk factors (23). A lower age at diagnosis of hearing loss in children with risk factors compared with those without risk factors was also reported by Robertson and colleagues. In Jafari's study in Iran no difference was observed in the age at hearing loss diagnosis and amplification between children with and without risk factors (19). In spite of a reduction in the age at hearing loss diagnosis in children with a family history of congenital sensorineural hearing loss, a lack of other factors necessary to perform intervention services, such as financial support, may result in a delay in cochlear implantation for these children. The age at cochlear implantation is not affected by the number of family members. However, where parents have a higher level of education the age at cochlear implantation is lower. In a study by Ozcebe and colleagues a low level of parental knowledge was considered to be a reason for delay in diagnosis of hearing loss (3). Robertson and colleagues indicated that the successfulness of a screening program depends on the attitude and knowledge of parents toward hearing loss (4). Perhaps higher levels of education lead to increased knowledge about symptoms and the effects of hearing loss, which in turn results in earlier referral to healthcare services and then earlier cochlear implantation.

Children who are part of families with better socioeconomic circumstances received cochlear implantation earlier. A study by Jafari and colleagues reported that the ages at diagnosis of hearing loss and intervention decrease in families who live in favorable socioeconomic circumstances compared with those who live in difficult circumstances (19). Similarly, Ozcebe and colleagues pointed to poor socioeconomic circumstances as an important factor in delay in identification of hearing loss and increases in the time lapse between amplification and intervention (3). Even though some families are immediately referred to a cochlear implantation center after diagnosis of hearing loss, lack of financial resources may be delay the occurrence of cochlear implantation. 

This study identifies the importance of the availability of adequate diagnostic and rehabilitative services for families and the worth of elevating public awareness about hearing loss and its treatment process for skills development in deaf children.

## Conclusion

Our study focused on the age at cochlear implantation and the effect of parents’ levels of education and economic circumstances on this age in a group of children with profound sensorineural hearing impairment. A remarkable delay between the ages of the children at each of the studied stages (suspicion, diagnosis, amplification, aural rehabilitation, and cochlear implantation) was observed. Also, parents’ levels of education and economic circumstances had a remarkable effect on the age at cochlear implantation. Our results highlight the importance of age at diagnosis of hearing loss and its effects on age at cochlear implantation, since early implantation has substantial effects on the improvement of communication skills in deaf children. More importantly, the main clinical point of this study is that it is possible to minimize the age at cochlear implantation via increasing the level of public knowledge regarding hearing loss, cochlear implantation, and providing support services for families. 
